# Plasticity and Susceptibility of Brain Morphometry Alterations to Insufficient Sleep

**DOI:** 10.3389/fpsyt.2018.00266

**Published:** 2018-06-27

**Authors:** Xi-Jian Dai, Jian Jiang, Zhiqiang Zhang, Xiao Nie, Bi-Xia Liu, Li Pei, Honghan Gong, Jianping Hu, Guangming Lu, Yang Zhan

**Affiliations:** ^1^Department of Medical Imaging, Medical School of Nanjing University, Jinling Hospital, Nanjing, China; ^2^Department of Radiology, The First Affiliated Hospital of Nanchang University, Nangchang, China; ^3^Department of Radiology, Yiyang Central Hospital, Yiyang, China; ^4^Department of ICU, Jiangxi Cancer Hospital, Nanchang, China; ^5^Brain Cognition and Brain Disease Institute, Shenzhen Institutes of Advanced Technology, Chinese Academy of Sciences, Shenzhen, China

**Keywords:** insomnia, sleep deprivation, voxel-based morphometry, gray matter, attention network test, spatial working memory

## Abstract

**Background:** Insufficient sleep is common in daily life and can lead to cognitive impairment. Sleep disturbance also exists in neuropsychiatric diseases. However, whether and how acute and chronic sleep loss affect brain morphology remain largely unknown.

**Methods:** We used voxel-based morphology method to study the brain structural changes during sleep deprivation (SD) at six time points of rested wakefulness, 20, 24, 32, 36 h SD, and after one night sleep in 22 healthy subjects, and in 39 patients with chronic primary insomnia relative to 39 status-matched good sleepers. Attention network and spatial memory tests were performed at each SD time point in the SD Procedure. The longitudinal data were analyzed using one-way repeated measures ANOVA, and *post-hoc* analysis was used to determine the between-group differences.

**Results:** Acute SD is associated with widespread gray matter volume (GMV) changes in the thalamus, cerebellum, insula and parietal cortex. Insomnia is associated with increased GMV in temporal cortex, insula and cerebellum. Acute SD is associated with brain atrophy and as SD hours prolong more areas show reduced GMV, and after one night sleep the brain atrophy is restored and replaced by increased GMV in brain areas. SD has accumulative negative effects on attention and working memory.

**Conclusions:** Acute SD and insomnia exhibit distinct morphological changes of GMV. SD has accumulative negative effects on brain morphology and advanced cognitive function. The altered GMV may provide neurobiological basis for attention and memory impairments following sleep loss.

**Statement of significance:**

Sleep is less frequently studied using imaging techniques than neurological and psychiatric disorders. Whether and how acute and chronic sleep loss affect brain morphology remain largely unknown. We used voxel-based morphology method to study brain structural changes in healthy subjects over multiple time points during sleep deprivation (SD) status and in patients with chronic insomnia. We found that prolonged acute SD together with one night sleep recovery exhibits accumulative atrophic effect and recovering plasticity on brain morphology, in line with behavioral changes on attentional tasks. Furthermore, acute SD and chronic insomnia exhibit distinct morphological changes of gray matter volume (GMV) but they also share overlapping GMV changes. The altered GMV may provide structural basis for attention and memory impairments following sleep loss.

## Introduction

We spend a third of our lives in sleep, yet sleep is less frequently studied using imaging techniques than many neurological and psychiatric disorders. Sleep is increasingly found to have far more health impact than what was previously thought. The precise control of sleep process is the basis of normal life process such as blood, metabolism, immune, endocrine, brain activity, and is the key of plasticity forming, information processing and function implementation (1–4). Sleep deprivation (SD) is associated with a series of maladaptive changes in alertness, judgment, emotion, memory, learning, immunity and central nervous system (5–12). Short-time SD may influence the expression of certain genes (13) while long-term SD can result in genetic changes (14). Insomnia as a general sleep disorder affects nearly 10–15% of the adult population (15). Insufficient sleep can lead to cognitive impairment, emotional change, brain dysfunction, psychomotor retardation and metabolic dysregulation (7, 8, 10, 12, 16–21). Despite the adverse socioeconomic impact of insufficient sleep, its neurobiological substrates are still elusive. Evidence suggests that chronic insomnia is accompanied by brain structural and functional changes (20–31). Elucidating brain-morphological changes of insufficient sleep can gain insights on the cognitive and emotional impacts by the sleep loss and bridges the gap between insufficient sleep and neurological or psychiatric disorders. Although SD is a frequently used protocol to investigate the functional consequences and behavioral changes associated with sleep loss (32, 33), what the brain structure changes temporally during the course of acute SD and what the brain structure changes after SD compare to those in patients with chronic primary insomnia remains unknown.

Sleep is associated with increased brain expression of genes involved in regulating macromolecule biosynthesis (34–37), and elevated transcription of genes involved in synthesis and maintenance of cell membrane lipids and myelin in the brain (34, 38, 39). Nevertheless, these structures might be particularly susceptible to insufficient sleep (39, 40). Recently a emerging view that structural brain gray matter and white matter changes can be observed within brief periods of time, from hours to days, following short-term learning (41) or neurotransmitter blockade (42). SD was associated with disturbed level of neurotransmitters (43), neuropeptides (44) and various kinds of cytokines (45) in the brain. In the longer term, rodent studies have shown that chronic sleep restriction and chronic stress are associated with brain structural changes (46, 47). The reported structural changes reflect the underlying pathology of the disease and may determine clinical phenomenology (48). Given the neurochemical changes by the SD and the link between the brain morphology and the neurochemical manipulation, we tested whether the brain structures exhibit changes as a result of insufficient sleep. First, we asked whether SD at different length of time could contribute to the changes in brain morphometry and its plasticity. Second, we examined the brain morphometry in patients with insomnia to understand whether short-term and chronic sleep loss may underlie shared structural basis.

Previous studies suggest that the reported structural changes reflected the underlying pathology of the disease and may determine clinical phenomenology (48). Voxel-based morphometry (VBM) method uses refined image registration and segmentation and provides sensitive measurements on the structural gray matter and white matter changes (49–51). In this study we applied VBM method to explore the dynamic evolution procedure of whole brain morphometry changes in the longitudinal data of 36-h acute SD and in a large sample of patients with insomnia. In the 36 h SD procedure, we performed repeated MRI sessions at 20, 24, 32, and 36 h after the SD started. We also performed one MRI session before the SD started and another one after one-night sleep recovery. Along with each MRI session, attentional network test (ANT) and spatial working memory task (SWM) were performed to evaluate the cognitive vulnerability to SD. In the insomnia study, we collected MRI data from patients with insomnia together with good sleepers (GSs).

## Materials and methods

### Subjects

This study was approved by the Medical Research Ethical Committee of Jinling Hospital and the First Affiliated Hospital of Nanchang University in accordance with the Declaration of Helsinki. The MRI and behavioral data were collected from two studies with a total of 100 subjects including an acute SD study and a chronic insomnia study. In the acute SD study, a total of 22 healthy university students (13 female, 9 male; mean age, 21.91 ± 1.38 years, mean ± standard deviation) participated an experiment of 36h SD design. In the chronic insomnia study, 39 patients with chronic primary insomnia (29 female, 10 male; mean age 48.92 ± 11.38 years, mean ± standard deviation) and 39 age-, sex-, and education-matched GSs (26 female, 13 male; mean age, 47.87 ± 9.15 years, mean ± standard deviation) were recruited. All volunteers participated voluntarily and were informed of the purposes, methods, and potential risks of this study, and signed an informed consent form. Twenty-one patients with insomnia (4 males, 17 females) were not the first-time visitors and previously had taken hypnotic or psychoactive medications. The other eighteen patients with insomnia (6 males, 12 females) were drug-naive and had never taken any medications before. The medication history duration was 1 month to 5 years. To avoid the possible effect of the medications, the patients with insomnia were kept medications-free for at least 2 weeks prior to the data collection and for the duration of this study, except that three patients with insomnia were medications-free for only 2–4 days. The mean duration of insomnia for patients with insomnia was 6.52 ± 5.65 (years, mean ± standard deviation).

Patients with insomnia met the relevant diagnostic criteria of the International Classification of Sleep Disorders, Second Edition(52), duration of insomnia > 1 year, Pittsburgh Sleep Quality Index (PSQI) score > 5, and sleep diary for >2 weeks duration. Furthermore, they had to report a total sleep time ≤ 6.5 h and (a) sleep onset latency > 45 min or (b) wake after sleep onset > 45 min or (c) total wake time during the sleep period (sleep latency + wake after sleep onset) > 60 min. To evaluate their sleep status, all subjects were asked to wear a Fitbit Flex tracker (http://help.fitbit.com) (20). These data were primarily used to verify sleep-wake diary information and not for independent assessment of inclusion and exclusion criteria.

All GSs and the 22 healthy university students met the following criteria: good sleeping habits, good sleep onset (<30 min) and/or maintenance (without easily wakened or morning awakening symptom) and regular dietary habits as measured by the Fitbit Flex tracker and sleep diary; no consumption of any stimulants, hypnotic or psychoactive medication, during or prior to the study for ≥3 months; PSQI score < 5, and Hamilton Depression Rating Scale (HAMD) and Hamilton Anxiety Rating Scale (HAMA) < 7. All subjects were right-handed. The exclusion criteria for all subjects comprised pathological brain magnetic resonance imaging (MRI) findings; inborn or other acquired diseases; any foreign implants in the body; BMI >32 or <19.8; present or past psychiatric or neurological disorders, substance dependency or substance abuse (including heroin, nicotine, or alcohol addiction); foreign implants in the body; any history of swing shift, night shift, or other shift work within the preceding year; any history of sleep complaints, or other sleep disorders, including hypersomnia, parasomnia, sleep related breathing disorder, sleep related movement disorder, or circadian rhythm sleep disorder, confirmed by overnight polysomnography; any history of significant head trauma or loss of consciousness >30 min; current smoking of more than 10 cigarettes per day; and consumption of >2 caffeinated beverages or potent tea per day.

### SD procedure

**Figure 1:**
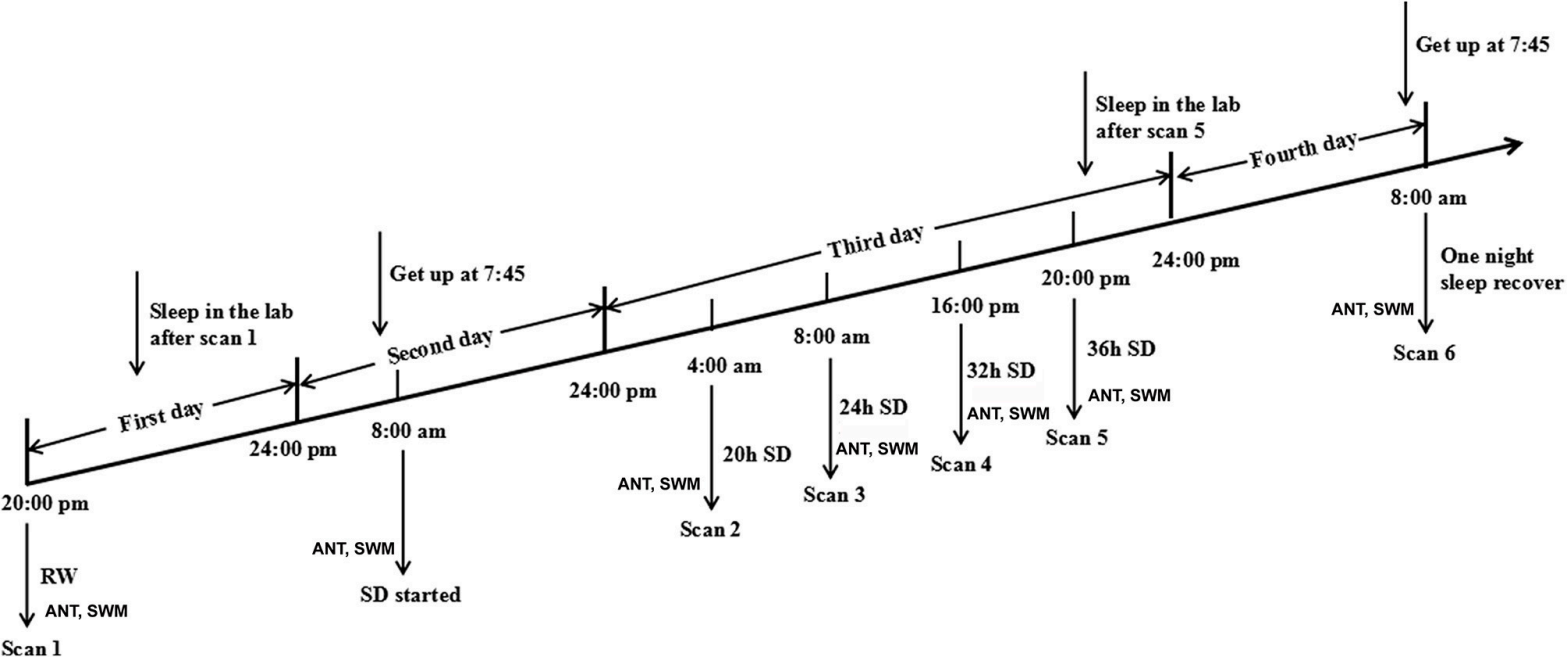
Schematic diagram of sleep deprivation (SD) procedure.

In the acute SD study, the SD Procedure started from 20:00 in the first day and lasted until 8:00 in the fourth day (Figure 1). All subjects were asked to arrive the lab at 19:00 in the first day (the day before the SD process) and underwent an MRI session as a base-line. All subjects were asked to sleep in the laboratory at the same time as usual. During this process, the subjects who had poor sleep quality were excluded. The 36 h SD Procedure started at 8:00 in the morning in the second day and lasted until 20:00 in the third day. The participants were required to stay awake during the entire time of the SD procedure. All subjects were not allowed to lie down and do some vigorous exercise, and they were not allowed to continue to do one thing for a long time, such as read and talk about an exciting topic. The food and water were provided during the SD procedure. Specially, all subjects eat the same food at every meal during the SD procedure to control the food intake, but the water are not controlled. The temperature of the room was maintained between 23 and 27°C. The staffs of the research team took charge of monitoring in turns through video monitors to make sure that the participants did not fall asleep during the SD procedure. If there were any signs of falling asleep, the participants were awakened by an alarm clock immediately.

Each subject underwent MRI sessions at the following time points: the start of the experiment during rested wakefulness (RW), 20, 24, 32, and 36 h after the experiment started (Figure 1). The subjects then spent one night of sleep for recovery and underwent another MRI session at 8:00 in the next morning in the fourth day. Furthermore, each subject underwent the long-term task of ANT and short-term simple task of SWM at each measurement time point before each of those MRI scans.

### Insomnia procedure

An experienced psychiatrist evaluated the patients with insomnia with the Diagnostic and Statistical Manual of Mental Disorders, version 4 (DSM-IV) (53) for the life history of psychiatric disorders, as well as an unstructured clinical interview for the history of medicine and sleep disorders. The patients with insomnia and status-matched GSs were asked to complete a number of questionnaires, including PSQI (54), Insomnia Severity Index (ISI) (55), Self-Rating Depression Scale (SDS) (56), Self Rating Anxiety Scale (SAS) (57), HAMD (58), HAMA (59), Self-Rating Scale of Sleep (SRSS) and Profile of Mood States (POMS) (60). The POMS questionnaire contains of 7 indexes, including 5 negative emotion indexes (nervousness, wrath, fatigue, depression and confusion) and 2 positive emotion indexes (energy and self-esteem). Then the patients with insomnia and the status-matched GSs each underwent the MRI scan once between 19:00 and 20:00.

### Attention network test (ANT)

The ANT, adapted from Fan et al.'s study (61, 62), contains of three cue conditions (no cue, center cue, spatial cue) and two target conditions (congruent and incongruent). The visual stimuli consisted of a row of 5 horizontal black arrows pointing leftward or rightward with the target arrow in the center. The participants responded to the direction of central arrow by pressing the left or right buttons of the computer mouse. The task measures alerting, orienting and conflict effects by calculate time difference between the response time and the presentation time under three different cue conditions.

The accuracy rate using corrected recognition, reaction time using only trials with correct responses, and lapse rate using missing recognition, were calculated. Finally, the intraindividual coefficient of variation was calculated for each participant by dividing the mean value of accuracy rate or correct reaction time by that of standard deviation.

### Spatial working memory test (SWM)

**Figure 2:**
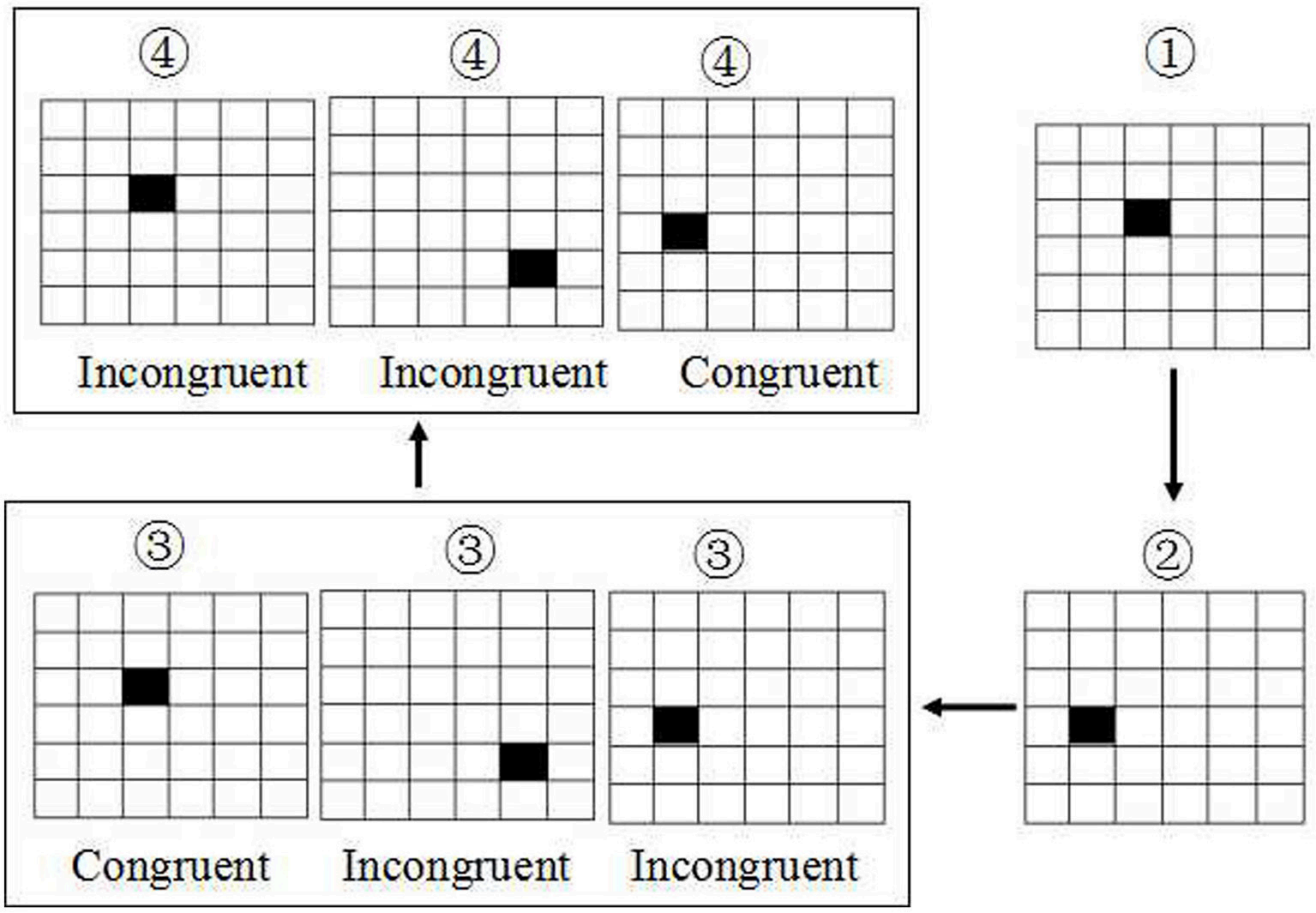
Schematic diagram of spatial working memory. The task was based on visual recognition of a series of 6 × 6 smaller squares with white. The first, second and third small square were filled with black respectively. Then the fourth square will be filled with black immediately once the first black square was recovered to white. At this time, the subjects were asked to make a keypress response to determine whether the location of the fourth black square was the same with the first black square, or subsequent the fifth was in the same location with the second black square, and so on.

The SWM was based on visual recognition of a series of 6 × 6 smaller squares filled in a large square with size of 7.2^*^7.2 mm^2^ (Figure 2). All these 36 small squares were filled with white. First, there was only shown a single small square filled with black in one location among these 36 smaller square. Next, the second and third small square was filled with black in another location respectively. Then, the fourth square will be filled with black immediately once the first small black square was recovered from black to white. At this time, the subjects were asked to make a keypress response to determine whether the location of the fourth black square was in the same location with the first black square, or subsequent the fifth black square was in the same location with the second black square, and so on. If they are in the same location, the subjects were asked to press the right button, conversely, the left button was conducted. The accuracy rate using corrected recognition, reaction time using only trials with correct responses, and lapse rate using missing recognition, were calculated. Finally, the intraindividual coefficient of variation was calculated for each participant by dividing the mean value of accuracy rate or correct reaction time by that of standard deviation.

### MRI parameter

The MRI scan was performed on a 3-Tesla MR scanner (Trio, Siemens, Erlangen, Germany). High-resolution T1-weighted anatomical images were acquired with a three-dimensional spoiled gradient-recalled sequence in sagittal orientation: 176 images (repetition time = 1,900 ms, echo time = 2.26 ms, thickness = 1.0 mm, gap = 0.5 mm, acquisition matrix = 256 × 256, field of view = 250 × 250 mm, flip angle = 9^0^) were obtained. A simple questionnaire was administered immediately after the ~3-min MRI scan to ask whether the subjects were awake during the scan. The data of subjects who fell asleep during the scan were excluded.

### Voxel-based morphometry (VBM)

MRIcro software (www.MRIcro.com) was used to ensure data quality. The data pre-processing was conducted using the available CAT12 toolbox (http://dbm.neuro.uni-jena.de/cat12/) which is based on Statistical Parametric Mapping 12 (SPM12, http://www.fil.ion.ucl.ac.uk/spm). First, the Digital Imaging and Communications in Medicine (DICOM) standard images were transformed into NIFIT format and were realigned into sagittal orientation. The images were corrected for bias field inhomogeneity by linear (12-parameter affine) and nonlinear transformations. Next, the structural images were segmented into gray matter, white matter, and cerebrospinal fluid (CSF). Diffeomorphic Anatomical Registration Through Exponentiated Lie algebra (DARTEL) segmentation procedure was performed in the present study. The 36 h SD Procedure were analyzed using segment procedures for the longitudinal data. The original unwrapped individual gray matter and white matter segmentations were warped to a newly constructed template with a combination of linear and nonlinear registration.

Then the data were spatially normalized using East Asian brain template to the Montreal Neurological Institute (MNI; http://www.mni.mcgill.ca/) space. The segmented data were modulated and smoothed using a Gaussian kernel of 8 × 8 × 8 mm^3^ full-width at half- maximum.

### Multiple linear regression analysis

Multiple linear regression analysis was performed to evaluate the relationships between the behavioral performance (dependent variable) in the ANT and SWM and the beta value of the main effect brain regions (independent variable) in each group of the 36 h SD study.

### Statistics

In the SD study, the behavioral data of the ANT and the SWM were analyzed using one-way repeated measures ANOVA. In the insomnia study, the demographic factors (age, education, and years of education) and the sleep questionnaire data were compared between the patients with insomnia and the GSs using two sample *t*-test. Chi-square (χ^2^) test was used for categorical data. The statistical analysis was performed using SPSS version IBM 21.0.

For the VBM data of the SD study, one-way within-subject repeated measures ANOVA was used to analyze the longitudinal data across the six time points during the 36 h process. The different brain regions of the main effect were saved as a mask. For the *post-hoc* analysis between two time points, we either calculated the product between the mask and the T maps or analyzed the difference without applying the mask.

For the VBM data in the insomnia study, unpaired *t*-test was used to investigate the gray matter volume (GMV) difference between the patients with insomnia and the GSs with the age, sex, years of education and total intracranial volume (TIV) as nuisance covariates of no interest.

We analyzed group differences in two ways. First, we used a threshold of *p* < 0.05, corrected for multiple comparisons by family-wise error (FWE) method. Second, we used an uncorrected statistical threshold of *p* < 0.001 with a minimum cluster size (k) of 100 voxels if the correction for multiple comparison failed to detect any difference.

## Results

### Main effect in 36 h SD study

**Figure 3:**
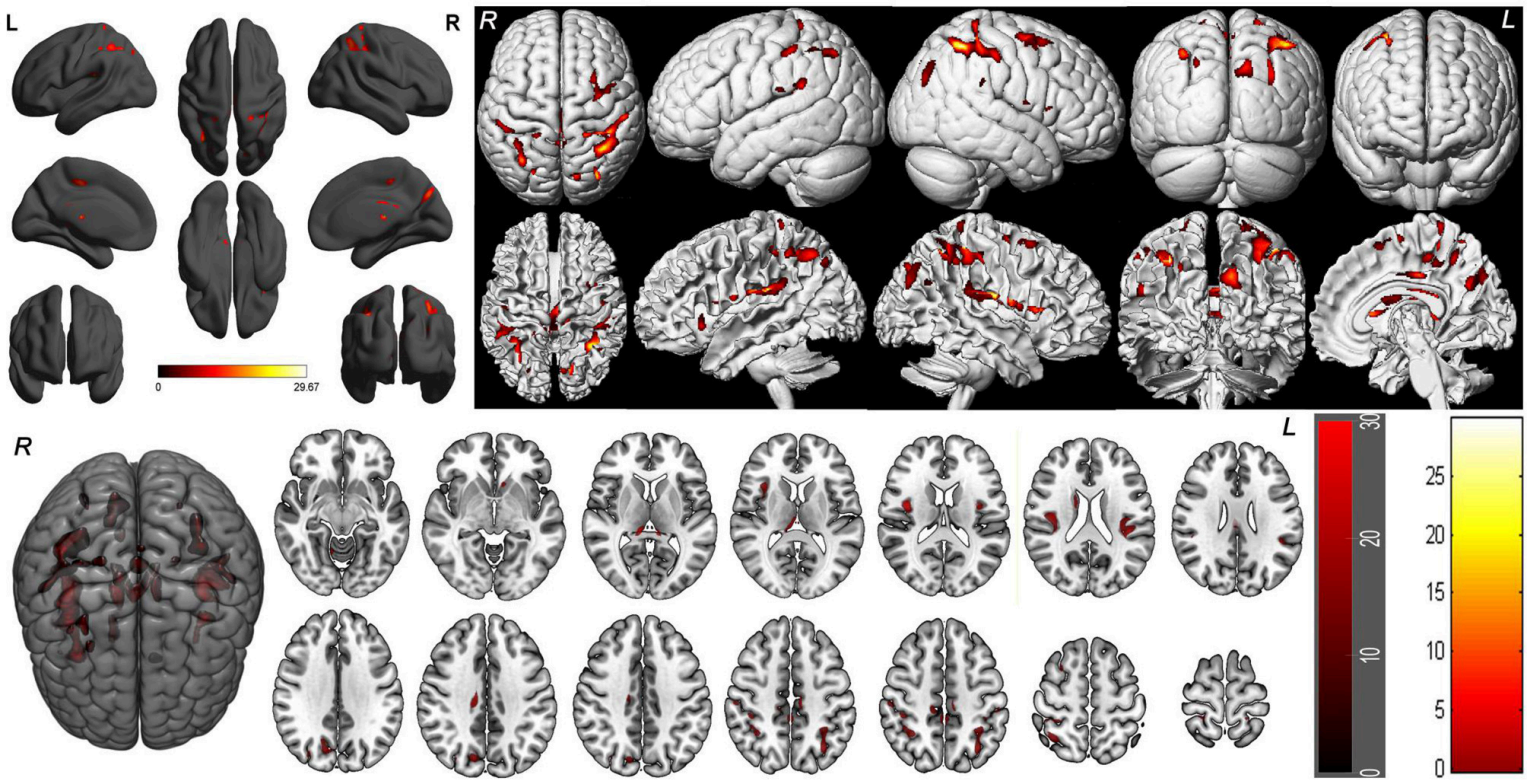
Brain-wide gray matter volume (GMV) differences of the 36 h sleep deprivation (SD) study. Main effect brain areas that passed the statistical criterion using the ANOVA test are marked in red color. Red areas denote positive main effect brain areas in GMV.

One-way within-subject repeated measures ANOVA with the SD time points as main factor revealed significant GMV differences in the right cerebellum anterior lobe, bilateral striatum (caudate), bilateral thalamus, bilateral insula (BA13), bilateral somatosensory association cortex (paracentral lobule, BA5; precuneus, BA7), bilateral somatosensory cortex (BA2), left superior parietal lobule (BA40), bilateral inferior parietal lobule (BA40), right supplementary motor area (SMA; BA6), bilateral posterior cingulate cortex extending to corpus callosum (BA23), and right cingulate cortex (BA24) [*F*_(5, 105)_ = 8.637, *p* < 0.05, *k* ≥ 100, corrected by FWE; Supplemental Table 1, Figure 3].

**Figure 4:**
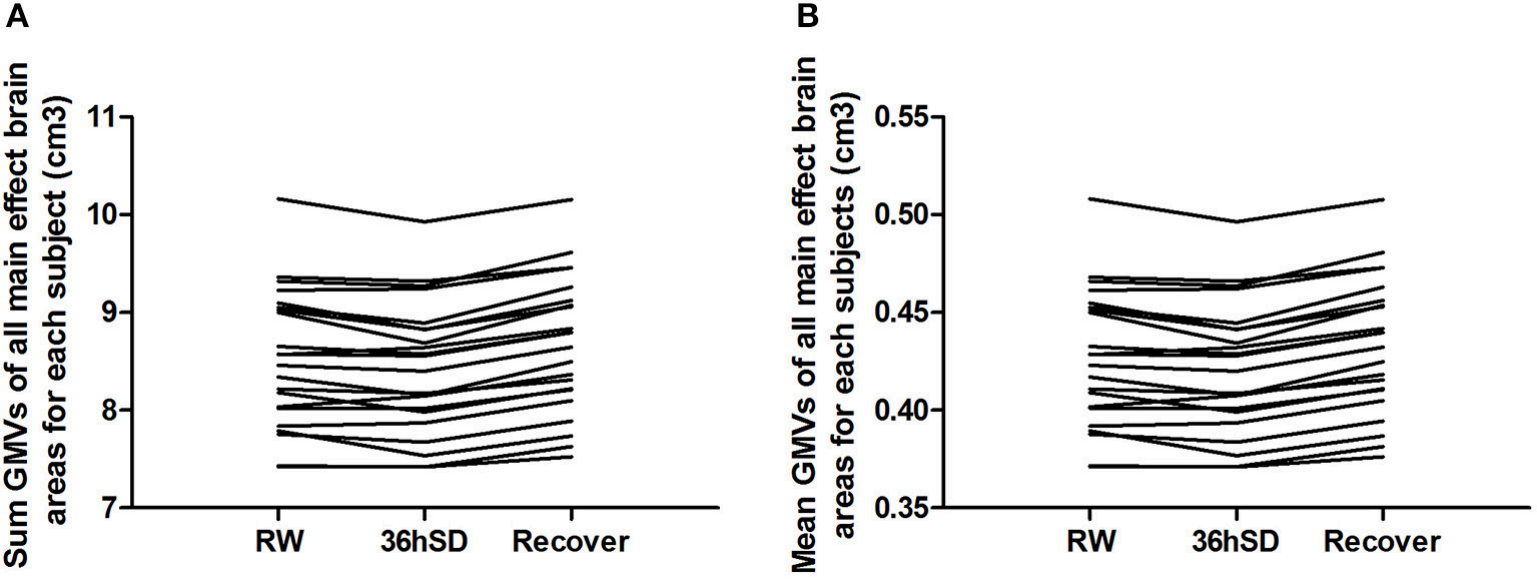
Sum and mean gray matter volume (GMV) of all main effect brain areas for each subject in the 36 h sleep deprivation (SD) study. In all subjects, from rested wakefulness (RW) to 36 h SD and from 36 h SD to one night sleep recovery, the total **(A)** and mean **(B)** GMV in the main effect brain areas showed a tendency of reduction first and then increase.

To account for the intra-individual differences, we then examined the beta values of the main effect areas from the ANOVA in each individual subject. Eighteen out of the 22 subjects (81.82%) exhibited smaller total and mean GMV at 36 h SD compared to RW, and the other 4 subjects showed increased GMV (in total 1.02% mean decrease). After one night sleep recovery 20 out of the 22 subjects (90.91%) showed larger GMV and the other two subjects showed decreased GMV (in total 1.75% mean increase) compared to RW. In all subjects, from RW to 36 h SD and from 36 h SD to one night sleep recovery, the total and mean GMV in the main effect brain areas showed a tendency of reduction first and then increase (Figure 4).

### *Post-hoc* tests in 36 h SD study

**Figure 5:**
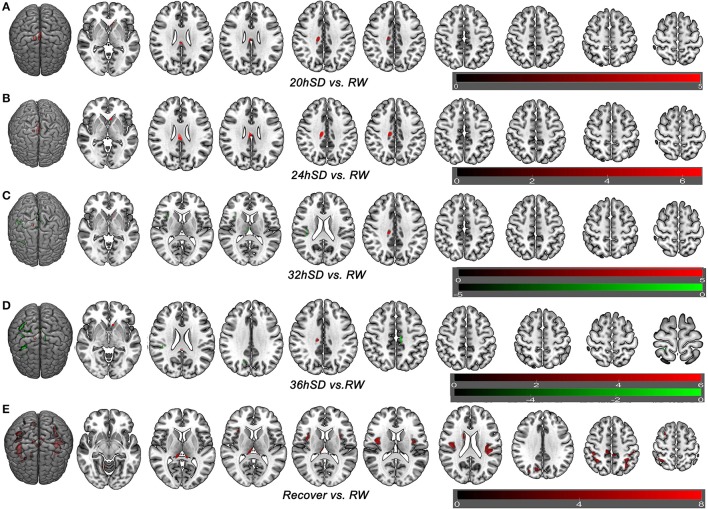
Brain-wide gray matter volume (GMV) differences of *post-hoc* test of each sleep deprivation (SD) time point in the 36 h SD study. The *post-hoc* test of each SD time point against RW was conducted as the product between the GMV differences of each time point and the GMV differences of main effect brain areas. Brain areas that showed GMV differences at each time point during the 36 h SD procedure against RW from the *post-hoc* tests, including the time point of 20 h SD **(A)**, 24 h SD **(B)**, 32 h SD **(C)**, 36 h SD **(D)**, and after one night sleep recovery **(E)**. Red areas denote increased GMV **(A–E)** and green areas denote decreased GMV **(C–D)** in brain areas.

To understand how SD at different length of time could contribute to the brain morphometry changes, we applied *post-hoc* tests to assess the GMV differences between various SD time points and the time point of RW using main effect brain regions as mask with an uncorrected statistical threshold (*p* < 0.001, *k* ≥ 100, uncorrected; Supplemental Table 2, Figure 5). A number of brain areas showed increased GMV at 20, 24, 32, and 36 h after the SD started, including the left striatum, right middle cingulate cortex (BA24) and bilateral posterior cingulate cortex extending to corpus callosum (BA23) (Figures 5A–D). On the other hand, a number of brain areas began to show decreased GMV at 32 h after the SD started, including the right thalamus, right insula (BA13), bilateral somatosensory association cortex (paracentral lobule, BA5; precuneus, BA7) and right inferior parietal lobule (BA40) (Figure 5C). Interestingly, 4 h later at 36 h after the SD started, the number of brain areas with decreased GMV increased, expanding to bilateral somatosensory cortex (BA2, BA3) and right SMA (BA6) (Figure 5D). After one night sleep recovery, no areas with decreased GMV were found but the right cerebellum anterior lobe, right striatum (caudate body), bilateral thalamus, bilateral insula (BA13), bilateral somatosensory association cortex (paracentral lobule, BA5; precuneus, BA7), bilateral inferior parietal lobule (BA40), bilateral somatosensory cortex (BA2), left superior parietal lobule (BA40) and right SMA (BA6) showed increased GMV (Figure 5E). Many of these brain areas with increased GMV (Figure 5E) were in the similar location as the areas showing decreased GMV at the SD time points relative to the time point of RW (Figures 5C,D), but they also contained more brain areas.

**Figure 6:**
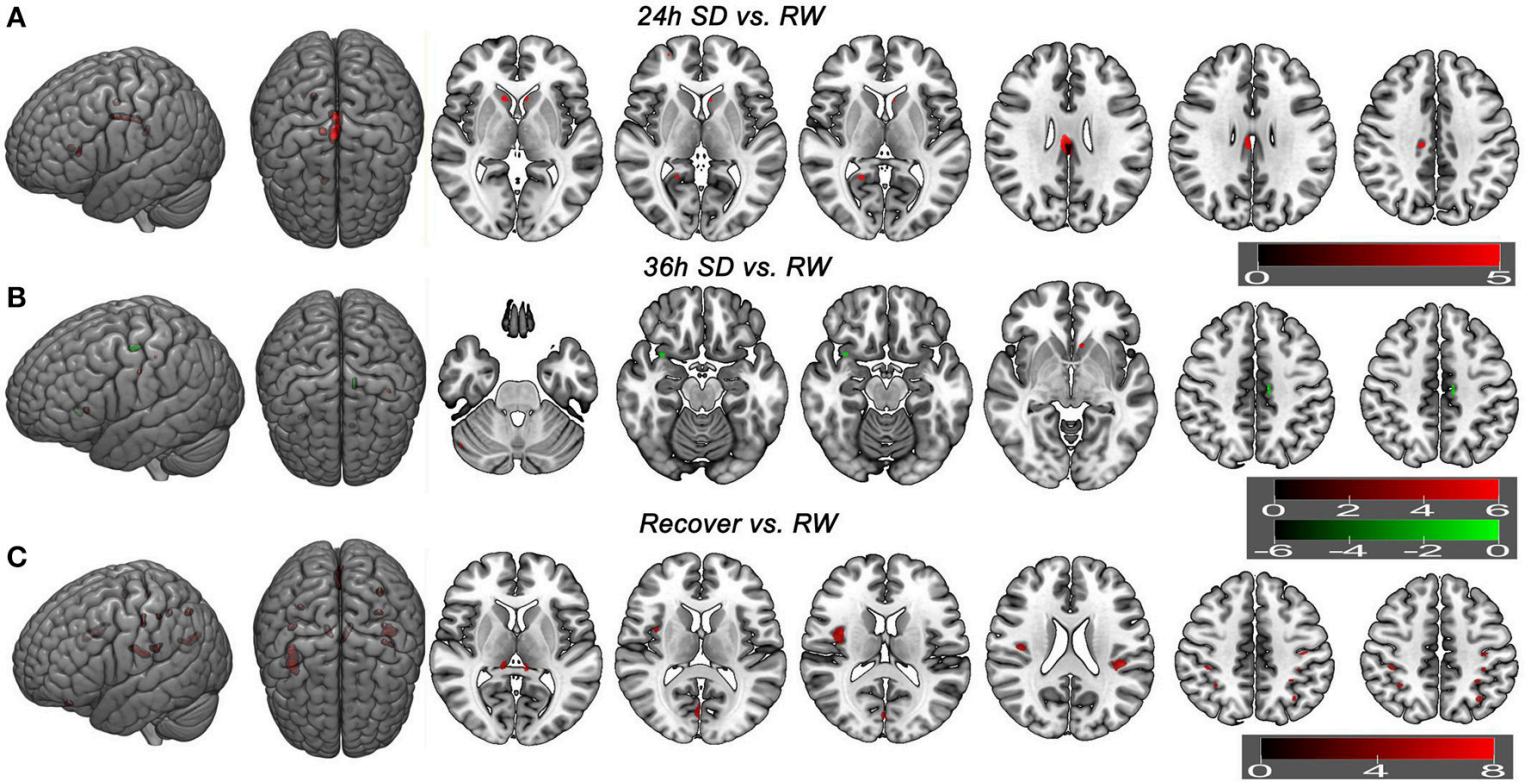
Brain gray matter volume (GMV) differences without applying mask method using *post-hoc t* test. Brain GMV differences were conducted using *post-hoc t*-test without applying the product with the mask of the different brain regions of main effect in 36 h sleep deprivation (SD) study. The statistical threshold was set at family-wise error corrected voxel threshold of *p* < 0.05 of each time in 36h SD study. The right side of the picture indicates the right side of the brain, and the corresponding left side indicates the left side of the brain. Red areas denote increased GMV brain regions **(A–C)** and green areas denote decreased GMV **(B)**. **(A)** Brain GMV differences at 24 h SD relative to rested wakefulness (RW). **(B)** Brain GMV differences at 36 h SD relative to RW. **(C)** Brain GMV differences after one night sleep recovery relative to RW.

Additionally we analyzed the GMV differences at each SD time point relative to the time point of RW using a corrected statistical threshold without applying the mask (*p* < 0.05, FWE corrected; Supplemental Table 3, Figure 6). This allowed to investigate the GMV changes on a broader scale. At 20 h after the SD started, VBM did not reveal any GMV difference relative to the RW. At 24 h after the SD started, bilateral striatum, bilateral cingulate gyrus (BA23), right posterior cingulate cortex (BA30) and right medial prefrontal cortex (BA10) showed increased GMV (Figure 6A). No decreased GMV was found. At 32 h after the SD started, only right cingulate gyrus (*t* = 5.0707; *x* = 13.5, *y* = −23.5, *z* = 36.5) showed increased GMV and no decreased GMV was found. At 36 h after the SD started, right cerebellum posterior lobe, left striatum and left somatosensory cortex (BA3) showed increased GMV (Figure 6B). The areas that showed decreased GMV included the left cingulate gyrus (BA24) and right temporal pole (BA38) (Figure 6B). After one night sleep recovery, bilateral thalamus, left orbitofrontal cortex (BA11), bilateral insula (BA13), right visual association cortex (BA18), bilateral somatosensory association cortex (BA7), bilateral parietal lobe (BA2, BA40) and left primary motor cortex (BA4) showed increased GMV (Figure 6C). No decreased GMV was found.

### Sample characteristics of patients with insomnia

The demographic characteristics of the patients with insomnia sample are presented in Table 1. There were no significant differences in sex distribution (*p* = 0.456), mean age (*p* = 0.654), mean education (*p* = 0.694) and PSQI time in bed (*p* = 0.725). However, compared with GSs, patients with insomnia showed shorter PSQI total sleep time, lower PSQI sleep efficiency, and higher PSQI score, higher SRSS score, higher SAS score, higher SDS score, higher HAMA score, higher HAMD score, higher POMS score, higher score of five negative index in POMS and lower score of two positive index in POMS (*p* < 0.001).

**Table 1:** Group characteristics of patients with insomnia and good sleepers.

	**Patients with insomnia**	**GSs**	***t*-value**	***p*-value**
**DEMOGRAPHICS**				
Mean age, year	48.92 ± 11.38	47.87 ± 9.15	0.45	0.654
Sex (male, female)	39 (10, 29)	39 (13, 26)	0.555^#^	0.456^#^
Education, year	6.95 ± 3.87	7.28 ± 3.58	−0.395	0.694
**SLEEP QUESTIONNAIRES**				
Duration of insomnia, year	6.52 ± 5.65	N/A	N/A	N/A
Pittsburgh Sleep Quality Index (PSQI)	15.05 ± 2.24	2.44 ± 0.88	32.781	< 0.001
PSQI total sleep time, hour	3.44 ± 1.24	7.38 ± 0.59	−17.965	< 0.001
PSQI time in bed, hour	8.36 ± 1.18	8.43 ± 0.48	−0.354	0.725
PSQI sleep efficiency, %	42.14 ± 17.24	87.34 ± 5.4	−15.622	< 0.001
Self Rating Scale Of Sleep (SRSS)	34.64 ± 4.77	15.92 ± 1.48	23.407	< 0.001
Insomnia Severity Index (ISI)	18 ± 3.01	N/A	N/A	N/A
Self-rating Anxiety Scale (SAS)	41.14 ± 8.69	27.69 ± 2.7	9.235	< 0.001
Self-Rating Depression Scale (SDS)	49.17 ± 9.78	31.26 ± 3.07	10.919	< 0.001
Hamilton Anxiety Scale (HAMA)	8.1 ± 3.7	1.85 ± 0.78	10.32	< 0.001
Hamilton Depression Scale (HAMD)	9.56 ± 4.89	2.26 ± 1.12	9.094	< 0.001
Profile of Mood States (POMS)	119.49 ± 21.22	83.08 ± 5.81	−9.701	< 0.001
Five negative index of POMS	31.67 ± 17.28	8.69 ± 3.25	8.16	< 0.001
Two positive index of POMS	12.38 ± 7.64	25.62 ± 3.77	10.333	< 0.001

# *chi-square value; Data are mean ± standard deviation values; GSs, Good sleepers; N/A, Not applicable; Self-rating Anxiety Scale and Self-Rating Depression Scale showed the standard score. The five negative index comprised nervousness, wrath, fatigue, depression and confusion, and the two positive index comprised energy and self-esteem*.

### VBM difference in patients with insomnia vs. GSs

There were no significant differences in the TIV, GMV, white matter volume (WMV), CSF volume, GMV/TIV, WMV/TIV, CSF volume (CSFM)/TIV and GMV/WMV between patients with insomnia and GSs (*p* > 0.05) (Table 2).

**Table 2:** Brain volume of patients with insomnia and GSs.

	**TIV (cm^3^)**	**GMV (cm^3^)**	**WMV (cm^3^)**	**CSFM Vol (cm^3^)**	**GMV/TIV(%)**	**WMV/TIV(%)**	**CSFM Vol/TIV(%)**	**GMV/WMV**
Patients with insomnia	1430.87 ± 123.04	618.33 ± 58.73	512.7 ± 52.37	299.85 ± 34.16	43.21 ± 1.61	35.8 ± 1.44	20.98 ± 1.92	1.21 ± 0.07
GSs	1455.37 ± 108.3	627.02 ± 43.37	517.92 ± 51.17	310.43 ± 46.46	43.16 ± 2.25	35.55 ± 1.65	21.29 ± 2.44	1.22 ± 0.09
*t*	−0.933	−0.744	−0.445	−1.146	0.121	0.714	−0.612	−0.42
*p*	0.354	0.459	0.658	0.255	0.904	0.478	0.542	0.676

*Data are mean ± standard deviation values; GSs, Good sleepers; TIV, Total intracranial volume; GMV, Gray matter volume; WMV, White matter volume; CSFM, Cerebrospinal fluid matter; Vol, Volume*.

**Figure 7:**
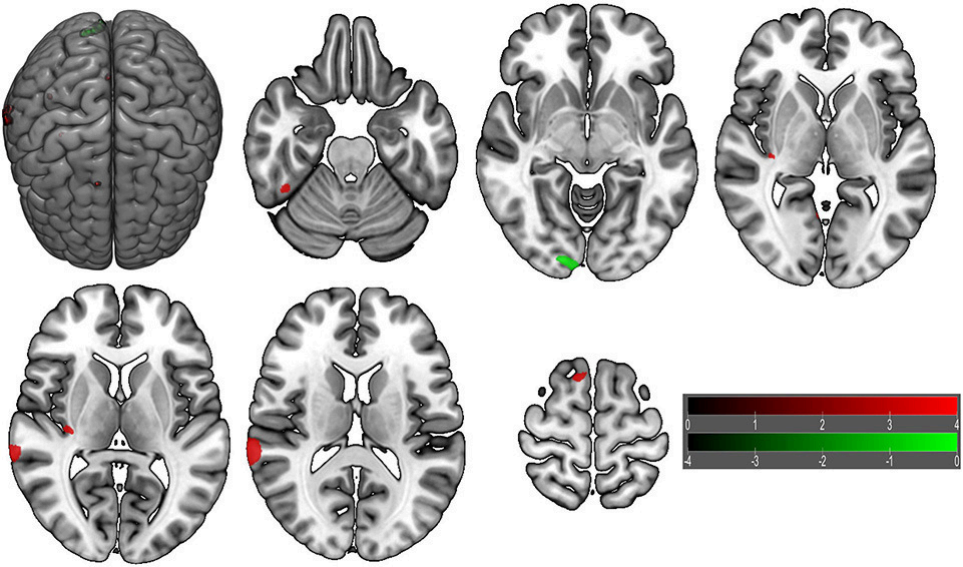
Brain gray matter volume (GMV) differences of patients with insomnia relative to good sleepers. The statistical threshold was set at uncorrected voxel threshold of *p* < 0.001 with a minimum cluster threshold of 100 voxels. The right side of the picture indicates the right side of the brain, and the corresponding left side indicates the left side of the brain. Green areas denote decreased GMV and red areas denote increased GMV.

VBM did not reveal any GMV difference between patients with insomnia and GSs using a two sample *t*-test (*p* < 0.05, FWE corrected). When using an uncorrected statistical criterion (*p* < 0.001, *k* ≥ 100), we found GMV differences localized in the right hemisphere (Supplemental Table 3, Figure 7), with increased GMV in the fusiform gyrus (BA 37), cluster of cerebellum anterior lobe and visual association cortex (BA18), cluster of claustrum and insula (BA13), primary auditory area (superior temporal gyrus, BA22, BA42) and SMA (BA 6), and with decreased GMV in the visual association cortex (BA18).

### Behavioral findings of 36 h SD study

**Figure 8:**
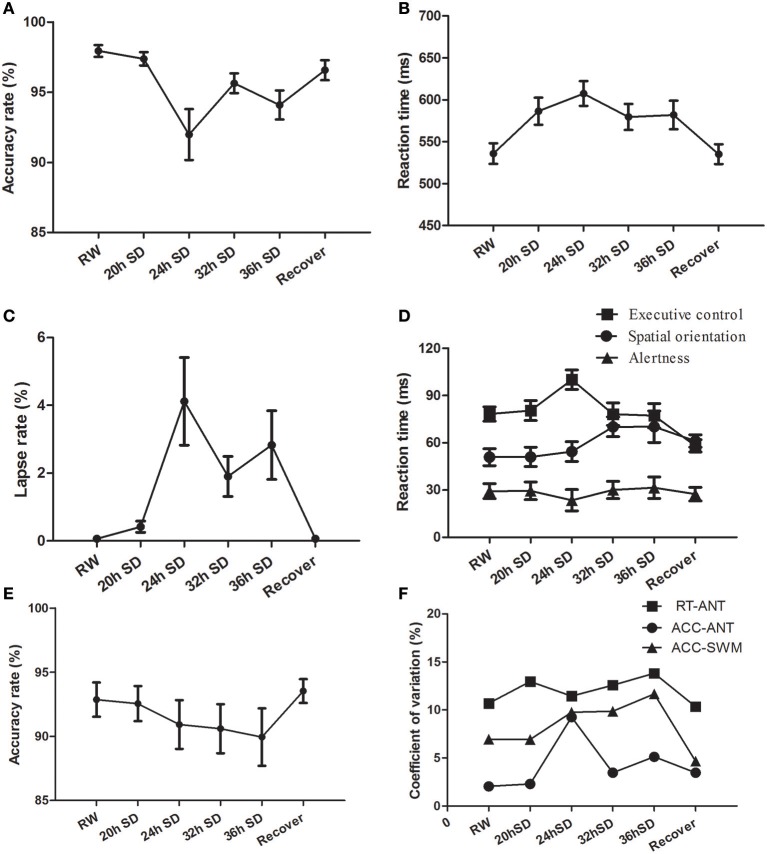
Behavioral findings of attention network test (ANT) and spatial working memory test (SWM). Each behavioral measurement was taken at six time points during the sleep deprivation (SD) session. **(A)** Accuracy rate of the ANT. **(B)** Reaction time of the ANT. **(C)** Lapse rate of the ANT. **(D)** Reaction time for the alertness, spatial orientation and executive control of the ANT. **(E)** Accuracy rate of the SWM. **(F)** Coefficient of variation values of three indexes, including reaction time of the ANT, accuracy rate of the ANT, and accuracy rate of the SWM. Data are presented as mean ± standard values.

We examined the attention and working memory in the ANT and SWM tests at different time points during the SD Procedure. The accuracy rate, reaction time and lapse rate of the ANT were different across the six SD time points using one-way repeated measures ANOVA [Greenhouse-Geisser correction, accuracy rate: *F*_(1.956, 41.072)_ = 8.299, *p* = 0.001, Figure 8A; reaction time *F*_(3.268, 68.631)_ = 11.242, *p* < 0.001, Figure 8B; lapse rate *F*_(1.975, 41.473)_ = 7.034, *p* = 0.002, Figure 8C]. The accuracy rate showed a tendency of gradual decrease (Figure 8A), and the reaction time and lapse rate showed a tendency of gradual increase as the SD hours prolonged (Figures 8B,C). The accuracy and reaction time restored after one night sleep recovery, but the accuracy rate and reaction time did not reach the level of RW completely. Interestingly, the subjects showed the lowest accuracy rate, longest reaction time and highest lapse rate at the time point of 24 h after the SD started. Furthermore, we measured the alertness, orienting and executive control from the ANT processes. The reaction time of spatial orientation and executive control were different across the SD time points [orienting: *F*_(5, 105)_ = 2.683, *p* = 0.025; executive control: *F*_(5, 105)_ = 6.003, *p* < 0.001; Figure 8D]. The reaction time of alertness was not different across the six time points [*F*_(5, 105)_ = 0.277, *p* = 0.925; Figure 8D]. In the SWM test, the accuracy rate did not show an effect of SD time [*F*_(5, 105)_ = 0.935, *p* = 0.461; Figure 8E], however there was a trend of gradual decrease as the SD hours prolonged and then a trend of increase after one night sleep recovery.

The intra-individual coefficient of variability for ANT accuracy rate, ANT reaction time, and SWM accuracy rate showed a tendency of increase as the SD hours prolonged and showed decrease after one night sleep recovery (Figure 8F). The accuracy rate of the ANT showed the highest intra-individual coefficient of variability at the time point of 24 h SD compared to other five time points.

### Intra-individual differences in behavior for each subject

We conducted the intra-individual differences in behavior as the intra-individual GMV differences in brain areas. We calculated the accuracy rate and reaction time for each subject (Supplemental Figure 1). In the ANT test, eighteen of the subjects shower lower accuracy rate, and the other four subjects showed higher accuracy rate at the time point of 36 h SD compared to RW (Supplemental Figure 1A). Twelve of the subjects demonstrated lower accuracy rate, two subjects showed no differences, and the other eight subjects indicated higher accuracy rate after one night sleep recover compared with RW (Supplemental Figure 1A). Seventeen of the subjects demonstrated longer reaction time, and the other five subjects showed shorter reaction time at the time point of 36h SD compared to RW (Supplemental Figure 1B). Ten of the subjects showed longer reaction time and the other twelve of the subjects indicated shorter reaction time after one night sleep recover compared with RW (Supplemental Figure 1B). In individual subjects, from RW to 36 h SD and from 36 h SD to one night sleep recovery, the accuracy rate in ANT showed a tendency of reduction first (36 h SD vs. RW, 3.94% mean decrease) and then increase (recovery vs. RW, 1.39% mean decrease), and the reaction time showed an inverse tendency of increase first (36 h SD vs. RW, 9.00% mean increase) and then decrease (recovery vs. RW, 0.05% mean increase) (Supplemental Figure 1).

In the SWM, nine of the subjects demonstrated lower accuracy rate, seven subjects showed no differences, and the other six subjects indicated higher accuracy rate at the time point of 36 h SD compared to RW (36 h SD vs. RW, 2.7% mean decrease) (Supplemental Figure 1C). Five of the subjects demonstrated lower accuracy rate, nine subjects showed no differences, and the other eight subjects indicated higher accuracy rate after one night sleep recover compared with RW (recovery vs. RW, 1.46% mean increase) (Supplemental Figure 1C). Eleven of the subjects demonstrated longer reaction time, and the other eleven subjects showed shorter reaction time at the time point of 36 h SD compared to RW (36 h SD vs. RW, 0.26% mean decrease) (Supplemental Figure 1D). Five of the subjects showed longer reaction time and the other seventeen of the subjects indicated shorter reaction time after one night sleep recover compared with RW (recovery vs. RW, 5.39% mean decrease) (Supplemental Figure 1D).

### Regression analysis between VBM and behavior

To investigate whether the structural changes during the SD status may have correlations with the behavior, we performed multiple linear regression between the behavioral parameters in the attention and working memory tasks and the beta values of the main effect brain areas at each SD time point (Table 3). Across the SD time points, the accuracy rate in the ANT and the SWM tests showed linear correlations with the beta value of many brain areas, including the somatosensory association cortex and insula. At the time points of RW and one night sleep recovery, linear relationships were found in the parietal lobe (somatosensory cortex and inferior parietal lobule).

**Table 3:** Multiple linear regression analysis between the beta value of main effect areas and behavioral findings.

**Group**	**Dependent variable**	**Independent variables**	**Coefficient (*R*^2^)**	**β**	**Standard error**	***t*-value**	***p*-value**
20hSD	Accuracy rate in ANT	Left precuneus	0.368	−0.351	0.103	−3.415	0.003
20hSD	Alertness in ANT	Left postcentral gyrus	0.275	−395.578	143.535	−2.756	0.012
20hSD	Executive control in ANT	Right insula	0.355	413.265	124.528	3.319	0.003
20hSD	Accuracy rate in SWM	Left Insula, Inferior parietal lobule	0.598	0.419	0.141	2.971	0.008
		Left superior parietal lobule, lnferior parietal lobule		0.387	0.170	2.269	0.036
		Left postcentral gyrus		−0.868	0.279	−3.109	0.006
20hSD	Reaction time in SWM	Right precuneus	0.272	−1158.708	423.6	−2.735	0.013
24hSD	Accuracy rate in ANT	Left paracentral lobule	0.304	−0.573	0.194	−2.954	0.008
24hSD	Alertness in ANT	Right caudate body	0.286	353.412	124.862	2.83	0.01
24hSD	Spatial orientation in ANT	Right insula	0.231	−330.661	135.044	−2.449	0.024
24hSD	Executive control in ANT	Right precuneus	0.557	−165.228	57.974	−2.85	0.01
		Right middle frontal gyrus		662.865	135.704	4.885	< 0.001
24hSD	Accuracy rate in SWM	Left insula	0.461	−1.191	0.328	−3.625	0.002
		Left Insula, Inferior Parietal Lobule		0.495	0.215	2.308	0.032
24hSD	Reaction time in SWM	Right precuneus	0.303	−1516.186	513.985	−2.95	0.008
32h SD	Alertness in ANT	Left Insula, Inferior parietal lobule	0.208	170.21	74.381	2.288	0.033
32h SD	Spatial orientation in ANT	Left precuneus	0.296	410.993	141.7	2.9	0.009
32h SD	Executive control in ANT	Right insula	0.369	565.979	170.759	3.314	0.004
		Precuneus, Paracentral lobule		−354.971	165.907	−2.140	0.046
32h SD	Accuracy rate in SWM	Left precuneus	0.48	−1.180	0.380	−3.106	0.006
		Right precuneus		0.442	0.155	2.850	0.010
32h SD	Reaction time in SWM	Right precuneus	0.585	−1834.941	360.138	−5.095	< 0.001
		Left precuneus		1520.079	508.598	2.989	0.008
36hSD	Accuracy rate in ANT	Right insula	0.426	−0.579	0.213	−2.725	0.013
		Right precuneus		0.340	0.098	3.484	0.002
36hSD	Lapse rate in ANT	Right precuneus	0.265	−25.945	9.656	−2.687	0.014
36hSD	Alertness in ANT	Left thalamus	0.184	−436.246	205.713	−2.121	0.047
36hSD	Accuracy rate in SWM	Right insula	0.42	1.962	0.534	3.676	0.002
		Left insula		−1.350	0.517	−2.612	0.017
36hSD	Reaction time in SWM	Right precuneus	0.246	−975.657	381.748	−2.556	0.019
Recover	Accuracy rate in ANT	Left postcentral gyrus, inferior parietal lobule	0.372	−0.363	0.105	−3.445	0.003
Recover	Reaction time in ANT	Left postcentral gyrus, inferior parietal lobule	0.239	−481.855	192.372	−2.505	0.021
Recover	Spatial orientation in ANT	Left paracentral lobule	0.317	−127.442	41.866	−3.044	0.006
Recover	Reaction time in SWM	Right precuneus	0.3	−966.936	330.269	−2.928	0.008

The alertness in the ANT showed linear relationships with the beta value of the striatum, parietal lobe, insula and thalamus across the SD time points, and the executive control in the ANT showed linear relationships with the beta value of the insula, somatosensory association cortex, parietal lobe and SMA. However, no brain areas showed correlations with the alertness or executive control at the time point of RW and one night sleep recovery. Interestingly, at the time point of 24 h SD, the alertness displayed linear correlation with the beta value of the striatum and the executive control showed linear relationship with the beta value of the SMA, and no correlation was found at other time points.

## Discussion

### Brain morphological changes during the acute SD

We found in our SD study that insufficient sleep is associated with widespread brain morphological changes. Although molecular basis for the microstructure-level changes requires further investigation, acute SD is associated with altered gene expression involved in macromolecule biosynthesis (34–37) in human studies and altered gene expression involved in cell membrane lipids and myelin in the mouse brain (34, 38, 39). The susceptibility of these cellular substrates to the rapid changes following sleep loss might contribute to the brain microstructure changes as we observed in our VBM analysis. In the animal studies, SD could lead to neuronal marker changes for apoptosis and morphology, and these changes were restored after sleep recovery (63, 64). Consistent with these patterns, our VBM data showed progressive structural atrophy as SD hours prolonged and these changes were restored and replaced by extensive and larger morphologic brain inflation after one night sleep recovery. Previous diffusion tensor imaging study showed that SD is associated with widespread fractional anisotropy decreases in several brain areas and as the waking prolonged the decreases become larger (65), which may associated with the reduced interstitial space volumes and increased resistance to water flux in the brain after waking than during sleep (66) and particularly susceptible of cell membrane lipids and myelin to insufficient sleep (39, 40). In our longitudinal data of 36 h SD procedure, the brain atrophy began to appear at 32 h SD and aggravated at 36 h SD. In agreement with the brain morphology, the accumulative negative effects were found in attention and spatial memory tests, but after one night sleep recovery they were restored incompletely showing a delayed recovery. We hypothesized that the VBM changes observed in the present study are more likely to be related to the changes in tissue hydration or other phenomena.

### Circadian rhythm influences during SD

A recent study has shown that the brain responses during the day and prolonged wakefulness showed circadian rhythmic patterns (67). Subcortical areas including the striatum and thalamus showed strong correlations with the melatonin levels and these areas showed increased responses when later hours in the day start. We also found increased GMV in the striatum starting at the time point of 20 h SD, and these increased GMV remained into the later stage of the SD. Evidence also indicates that individuals with late chronotype who prefer to go to bed late in the evening had structural differences in the cingulate cortex and corpus callosum (68). In our SD study, we also found increased GMV in the corpus callosum and cingulate cortex. Considering the similar brain areas found in our study and the others, the structural changes in our VBM analysis may reflect the influence of circadian rhythm.

At the time point of 24 h SD, the subjects exhibited the lowest accuracy rate, longest reaction time and highest lapse rate in the ANT compared to the other time points. Accordingly, at this time point a number of increased GMV areas in the bilateral striatum and bilateral cingulate cortex extending to corpus callosum were found, and these areas existed even after the FWE correction. Specifically, we found that at 24 h SD the alertness displayed linear correlation with the beta value of striatum and the executive control showed linear correlation with the beta value of SMA, and these relationships were not found at other SD time points. At this time point of 8:00 a.m. in the morning, the participants usually woke up in their daily schedule in the process of getting out of bed and they showed reduced alertness. The SMA area was implicated in sensory processing, working memory, executive control and spatial-bodily attention (69, 70). Therefore the structural changes we observed in our data may underlie the circadian rhythm and prolonged wakefulness to modulate the attentional performance.

### Acute SD vs. chronic insomnia

Chronic insomnia is thought to be maintained by excessive negatively toned cognitive activity with autonomic arousal and emotional distress (71). The exaggerated cortical and somatic activation can lead to increased sensory information processing and inability to initiate or maintain sleep (20, 72, 73), and may be a result of increased brain activities. We found that both in the SD study and the insomnia study that the insula and cerebellum showed increased GMV. This demonstrates that acute and chronic sleep loss may also share similar neurobiological representation in brain morphology. In the SD study after one night sleep recovery only brain areas with increased GMV were found. Similarly, patients with insomnia also mainly found morphological differences in brain regions with increased GMV. This demonstrates that the status of subjects who underwent SD process and then received one night sleep recovery may exhibit analogous brain activation characteristics to the status of patients with insomnia who underwent subjective experience of chronically disturbed and non-refreshing sleep.

Although using an uncorrected statistical threshold we found a number of brain areas with GMV changes in patients with insomnia, using a strict criterion we did not observe differences between patients with insomnia and GSs. This probably demonstrates that the patients with insomnia were not prone to the brain microstructure changes already. Under the less stringent method we found altered GMV in the patients with insomnia in the temporal cortex, primary auditory area, insula, SMA and visual association cortex. For the acute SD the main effect areas with GMV changes were showed in the sensory cortex, motor cortex and subcortical thalamus. Therefore the two types of short-term and chronic sleep losses mainly exhibited non-overlapping altered brain areas.

In the insomnia study, the superior temporal cortex with increased GMV contains the primary auditory area (BA42). Previous study has shown that normal activation of the auditory cortex is decreased to help maintain sleep in response to external stimuli (74). Therefore, our observation of increased GMV in the auditory cortex may highlight the reduced capacity to disengage from external information processing of auditory stimuli, which was consistent with the clinical characteristics of insomnia patients with shallow sleep and increased sensitivity to surrounding environments. Our data therefore support the theory of hyperarousal, which may be a core predisposing or perpetuating factor of ultimately hampering the ability to initiate or maintain sleep.

Previous meta-analytical data demonstrated that the threat or anxiety hypothesis is associate with insula (75), and the craving hypothesis is associated with ventral striatum and cingulated cortex (76). Patients with insomnia underwent prolonged experience of chronically disturbed and non-refreshing sleep, and may display threat or anxiety in response to sleep-related cues. Subjects who underwent acute SD process may mainly display craving to sleep but not threat or anxiety (77). Our previous neuroimaging studies also found that insufficient sleep resulted in abnormal regional brain activity in the threat-related brain areas and craving-related brain areas (8, 20, 21, 78–80). Our observation of increased GMV in the insula or cingulated cortex in the insomnia study and increased GMV in the striatum in the SD study might support the theory of threat and craving hypothesis.

The paracentral lobule is considered to be negatively correlated with vigor activity (81). It has been found that the inferior parietal cortex area is impaired after SD and may represent an most reliable early biological marker of individual resistance to SD (7, 8, 82, 83). The postcentral gyrus is the main receptive region for external stimuli as the location of the primary somatosensory cortex. Recently the postcentral gyrus was shown to be implicated with the default mode network (84), which is a functional brain hub showing coupled slow signal fluctuations in the absence of external stimuli during restful waking and sleep (85). In the SD study, these areas with decreased GMV were found with several correlations with the ANT and SWM. Our observations of decreased GMV in theses somatosensory areas after acute SD in individuals who showed a possible insufficiency to enter into “resting state” status may reflect inhibition in sensory-informational processing and difficulties in cognitive function.

## Conclusions

In summary, acute SD and insomnia showed widespread changes in gray matter microstructure with shared but also distinct neurobiological representation in brain morphology. Acute SD may be associated with inhibition in sensory-informational processing with decreased GMV in the somatosensory areas to compensate for the effects of sleep loss on advanced cognitive function, while the insomnia may be associated with inability to disengage from external information processing of auditory stimuli with increased GMV in the primary auditory area. Prolonged acute SD together with one night sleep recovery exhibit accumulative atrophic effect and recovering plasticity on brain morphology, in line with the behavioral changes on attentional and working memory tasks. Taken together, clarifying the biological underpinnings of these microstructural alterations could advance our understanding of the neurobiological mechanism of waking and sleep. One of the strengths of the present study is the relatively large sample size in the insomnia study and longitudinal data in the SD study; however, there are several limitations that should be noted. First, our findings are limited by the use of the Fitbit Flex tracker to monitor the sleep quality in our experience (20, 86). Although we cannot provide direct evidence to prove whether the FITBIT tracker provides a valid and reliable measure of objective sleep, we compared some patients' data between the FITBIT and the PSG, and found the results were similar. In fact, our sample was screened to exclude individuals with medical or psychiatric disorders that may affect sleep, and the diagnosis of primary insomnia mainly depends on the experience of senior physicians who have been working for more than 20 years. Second, the subjects were not monitored by continuous EEG in the SD procedure, but a simple questionnaire was administered immediately after the MRI scan to ask whether the subjects were awake during the scan. The data of subjects who fell asleep during the scan were excluded.

## Author contributions

X-JD and YZ wrote the main manuscript text. X-JD, JJ, LP, HG, GL, and YZ conceived and designed the whole experiment. X-JD, XN, and B-XL collected the data. X-JD, JH, and ZZ analyzed the data.

### Conflict of interest statement

The authors declare that the research was conducted in the absence of any commercial or financial relationships that could be construed as a potential conflict of interest.

## Supplementary material

Supplemental Figure 1 Accuracy rate and reaction time in the ANT and spatial working memory (SWM) for each subject in the 36 h sleep deprivation (SD) study. **(A)** Accuracy rate of the ANT for each subject. **(B)** Reaction time of the ANT for each subject. **(C)** Accuracy rate of the SWM for each subject. **(B)** Reaction time of the SWM for each subject. In all subjects, from rested wakefulness (RW) to 36 h SD and from 36 h SD to one night sleep recovery, the accuracy rate showed a tendency of reduction first and then increase, and the reaction time showed a tendency of increase first and then decrease **(A–D)**.

Supplemental Table 1 The gray matter volume differences of main effect in the 36 h sleep deprivation study. R, right; L, left; BA, Brodmann's area; MNI, montreal neurological institute; N/A, Not applicable. The statistical threshold was set at corrected voxel threshold of *p* < 0.05 with a minimum cluster threshold of 100 voxels, corrected by family-wise error.

Supplemental Table 2 The *post-hoc* differences of gray matter volume with the product with the mask of the different brain regions of main effect in the 36 h sleep deprivation (SD) study. RW, Rested wakefulness; R, right; L, left; BA, Brodmann's area; MNI, montreal neurological institute; N/A, Not applicable. The statistical threshold was set at uncorrected voxel threshold of *p* < 0.001 with a minimum cluster threshold of 100 voxels (*t* = 3.1697).

Supplemental Table 3 The gray matter volume differences without applying mask method in the 36 h sleep deprivation (SD) study and in the chronic insomnia study. RW, Rested wakefulness; R, right; L, left; BA, Brodmann's area; MNI, montreal neurological institute; N/A, Not applicable; GSs, good sleepers. The statistical threshold was set at family-wise error corrected voxel threshold of *p* < 0.05 of each time in the 36 h SD study without product with the mask image of main effect, and at uncorrected voxel threshold of *p* < 0.001 with a minimum cluster threshold of 100 voxels in patients with insomnia.

## Abbreviations

SD: Sleep deprivation
GMV: Gray matter volume
VBM: Voxel-based morphometry
ANT: Attentional network test
SWM: Spatial working memory
ROIs: Regions of interest
GSs: Good sleepers
RW: Rested wakefulness
PSQI: Pittsburgh Sleep Quality Index
HAMD: Hamilton Depression Rating Scale
HAMA: Hamilton Anxiety Rating Scale
DSM-IV: Diagnostic and Statistical Manual of Mental Disorders, version 4
ISI: Insomnia Severity Index
SDS: Self-Rating Depression Scale
SAS: Self Rating Anxiety Scale
SRSS: Self-Rating Scale of Sleep
POMS: Profile of Mood States
SPM12: Statistical Parametric Mapping 12
DICOM: Digital Imaging and Communications in Medicine
CSF: Cerebrospinal fluid
DARTEL: Diffeomorphic Anatomical Registration Through Exponentiated Lie algebra
MNI: Montreal Neurological Institute
TIV: Total intracranial volume
FWE: Family-wise error
WMV: White matter volume.
