# Supplementary figures and images for: Plasticity and Susceptibility of Brain Morphometry Alterations to Insufficient Sleep
Source: Front Psychiatry. 2018 Jun 27;9:266. doi: 10.3389/fpsyt.2018.00266 (PMC6030367; doi:10.3389/fpsyt.2018.00266)

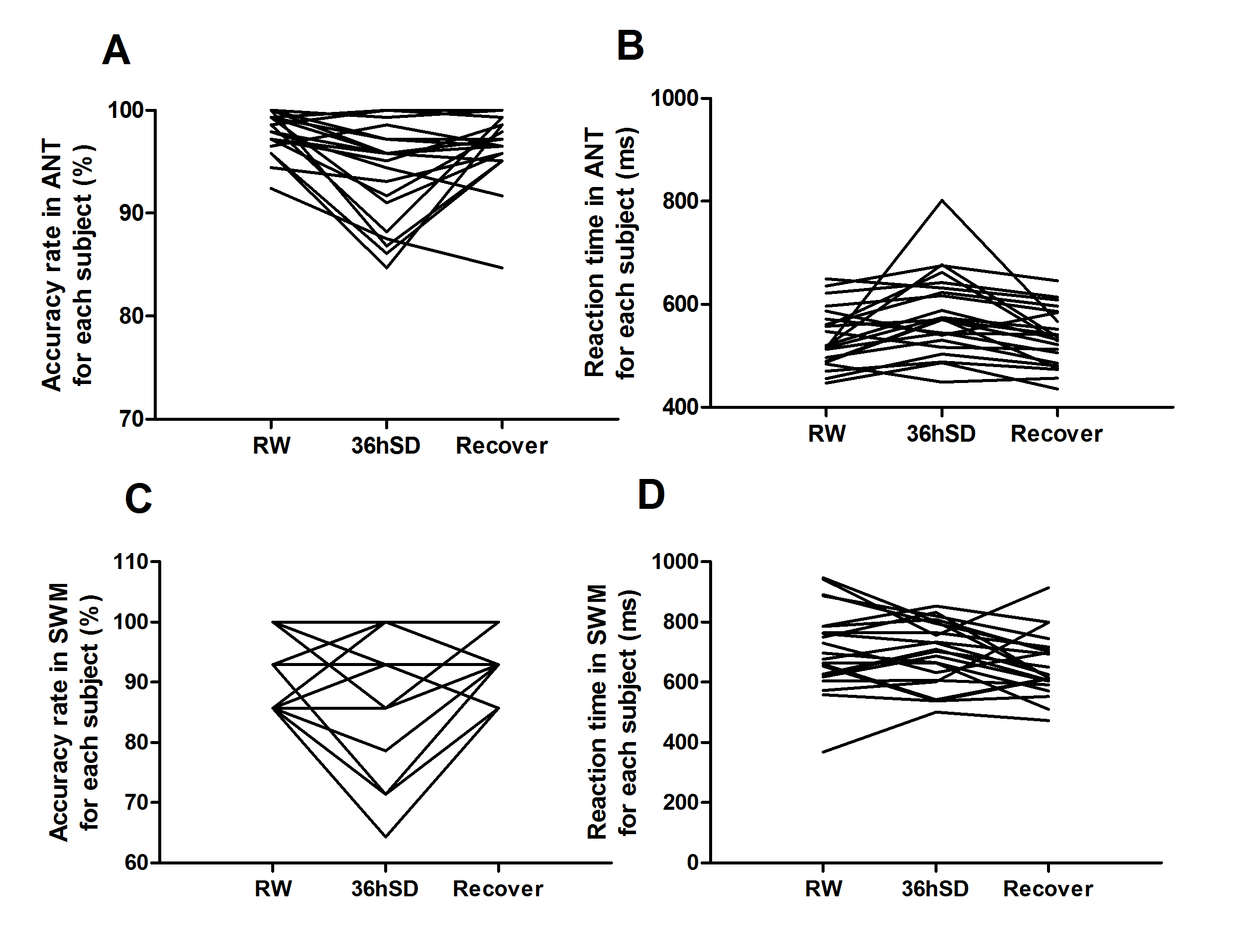

Supplement: Supplemental Figure 1 — Accuracy rate and reaction time in the ANT and spatial working memory (SWM) for each subject in the 36 h sleep deprivation (SD) study. (A) Accuracy rate of the ANT for each subject. (B) Reaction time of the ANT for each subject. (C) Accuracy rate of the SWM for each subject. (B) Reaction time of the SWM for each subject. In all subjects, from rested wakefulness (RW) to 36 h SD and from 36 h SD to one night sleep recovery, the accuracy rate showed a tendency of reduction first and then increase, and the reaction time showed a tendency of increase first and then decrease (A–D). [file Image_1.JPEG]
