# Supplementary material for: Plasticity and Susceptibility of Brain Morphometry Alterations to Insufficient Sleep
Source: Front Psychiatry. 2018 Jun 27;9:266. doi: 10.3389/fpsyt.2018.00266 (PMC6030367; doi:10.3389/fpsyt.2018.00266)
Supplement: Supplemental Table 1 — The gray matter volume differences of main effect in the 36 h sleep deprivation study. R, right; L, left; BA, Brodmann's area; MNI, montreal neurological institute; N/A, Not applicable. The statistical threshold was set at corrected voxel threshold of p < 0.05 with a minimum cluster threshold of 100 voxels, corrected by family-wise error. [file Table_1.DOC]

**Plasticity and susceptibility of brain morphometry alterations to insufficient sleep**

**Supplemental Information**

**Supplemental Table 1** The gray matter volume differences of main effect in 36h sleep deprivation study

| Brain regions of peak coordinates | R/L | BA | Voxel size | t-score of peak voxel | Peak MNI coordinates |
| --- | --- | --- | --- | --- | --- |
|  |  |  |  |  | X, Y, Z |
| Cerebellum Anterior Lobe | R | N/A | 237 | 13.7117 | 10.5 -56.5 -8.5 |
| Caudate Head | L | N/A | 271 | 16.2136 | -9.5 17.5 -4.5 |
| Caudate Body | R | N/A | 334 | 14.8068 | 13.5 -2.5 21.5 |
| Thalamus | L, R | N/A | 1289 | 22.0727 | -6.5 -33.5 8.5 |
| Insula | R | 13 | 2093 | 29.6664 | 39.5 -5.5 18.5 |
| Insula | L | 13 | 307 | 18.7211 | -38.5 -5.5 17.5 |
| Insula, Inferior Parietal Lobule | L | 13, 40 | 1346 | 26.491 | -39.5 -30.5 22.5 |
| Corpus Callosum, Posterior Cingulate Cortex | L, R | 23 | 280 | 11.0414 | 2.5 -23.5 25.5 |
| Cingulate Cortex | R | 24 | 328 | 21.8132 | 13.5 -21.5 36.5 |
| Precuneus | R | 19 | 454 | 12.7884 | 31.5 -77.5 31.5 |
| Precuneus | L | 7 | 129 | 10.5652 | -23.5 -73.5 42.5 |
| Precuneus | R | 7 | 1363 | 16.5251 | 11.5 -74.5 35.5 |
| Precuneus, Paracentral Lobule | L, R | 5,7 | 1000 | 14.4024 | -0.5 -38.5 48.5 |
| Paracentral Lobule | L | 5 | 178 | 10.4559 | -1.5 -47.5 62.5 |
| Paracentral Lobule | L | N/A | 294 | 15.3099 | -10.5 -22.5 47.5 |
| Superior Parietal Lobule, Inferior Parietal Lobule | L | 7,40 | 1428 | 17.9013 | -33.5 -63.5 47.5 |
| Postcentral Gyrus, Inferior Parietal Lobule | L | 2,40 | 463 | 11.7464 | -46.5 -35.5 52.5 |
| Inferior Parietal Lobule, Postcentral Gyrus | R | 2,7,40 | 3799 | 19.0754 | 41.5 -50.5 55.5 |
| Postcentral Gyrus | L | 2 | 120 | 10.8137 | -21.5 -41.5 69.5 |
| Middle Frontal Gyrus | R | 6 | 795 | 12.3656 | 32.5 -4.5 62.5 |

R, right; L, left; BA, Brodmann’s area; MNI, montreal neurological institute; N/A, Not applicable. The statistical threshold was set at corrected voxel threshold of p<0.05 with a minimum cluster threshold of 100 voxels, corrected by family-wise error.
