# Supplementary material for: Plasticity and Susceptibility of Brain Morphometry Alterations to Insufficient Sleep
Source: Front Psychiatry. 2018 Jun 27;9:266. doi: 10.3389/fpsyt.2018.00266 (PMC6030367; doi:10.3389/fpsyt.2018.00266)
Supplement: Supplemental Table 2 — The post-hoc differences of gray matter volume with the product with the mask of the different brain regions of main effect in the 36 h sleep deprivation (SD) study. RW, Rested wakefulness; R, right; L, left; BA, Brodmann's area; MNI, montreal neurological institute; N/A, Not applicable. The statistical threshold was set at uncorrected voxel threshold of p < 0.001 with a minimum cluster threshold of 100 voxels (t = 3.1697). [file Table_2.DOC]

**Plasticity and susceptibility of brain morphometry alterations to insufficient sleep**

**Supplemental Information**

**Supplemental Table 2** The post hoc differences of gray matter volume with the product with the mask of the different brain regions of main effect in 36h sleep deprivation (SD) study

| Conditions | Brain regions of peak coordinates | R/L | BA | Voxel size | t-score of peak voxel | Peak MNI coordinates |
| --- | --- | --- | --- | --- | --- | --- |
|  |  |  |  |  |  | X, Y, Z |
| 20hSD>RW | Caudate Head | L | N/A | 145 | 4.1156 | -8.5 17.5 -1.5 |
| 20hSD>RW | Corpus Callosum, Posterior Cingulate Cortex | L,R | 23 | 269 | 4.3291 | 2.5 -35.5 22.5 |
| 20hSD>RW | Cingulate Cortex | R | 24 | 255 | 4.8183 | 12.5 -21.5 37.5 |
| 24hSD>RW | Caudate Head | L | N/A | 156 | 5.4953 | -6.5 18.5 2.5 |
| 24hSD>RW | Corpus Callosum, Posterior Cingulate Cortex | L,R | 23 | 280 | 6.3082 | 2.5 -35.5 22.5 |
| 24hSD>RW | Cingulate Cortex | R | 24 | 252 | 5.7818 | 12.5 -22.5 36.5 |
| 32hSD>RW | Caudate Head | L | N/A | 253 | 4.3715 | -9.5 17.5 -3.5 |
| 32hSD>RW | Cingulate Cortex | R | 24 | 276 | 4.8183 | 13.5 -23.5 36.5 |
| 32hSD<RW | Thalamus | R | N/A | 121 | -4.1155 | 3.5 -19.5 13.5 |
| 32hSD<RW | Insula | R | 13 | 225 | -4.4078 | 32.5 14.5 11.5 |
| 32hSD<RW | Insula, Inferior Parietal Lobule | R | 13, 40 | 421 | -4.8681 | 40.5 -31.5 23.5 |
| 32hSD<RW | Precuneus, Paracentral Lobule | L,R | 5,7 | 221 | -4.0661 | -0.5 -36.5 47.5 |
| 32hSD<RW | Paracentral Lobule | L | 5 | 116 | -4.0188 | -11.5 -26.5 47.5 |
| 32hSD<RW | Paracentral Lobule | L,R | 5 | 159 | -3.7637 | 2.5 -51.5 62.5 |
| 36hSD>RW | Caudate Head | L | N/A | 227 | 5.5553 | -9.5 17.5 -4.5 |
| 36hSD>RW | Corpus Callosum, Posterior Cingulate Cortex | L,R | 23 | 198 | 4.4727 | 0.5 -36.5 22.5 |
| 36hSD>RW | Cingulate Cortex | R | 24 | 177 | 4.58 | 12.5 -22.5 36.5 |
| 36hSD<RW | Thalamus | R | N/A | 136 | -3.7474 | 11.5 -32.5 8.5 |
| 36hSD<RW | Insula | R | 13 | 143 | -3.9875 | 36.5 14.5 12.5 |
| 36hSD<RW | Insula, Inferior Parietal Lobule | R | 13, 40 | 406 | -4.7568 | 39.5 -31.5 22.5 |
| 36hSD<RW | Inferior Parietal Lobule, Postcentral Gyrus | R | 2,7,40 | 1838 | -4.7297 | 40.5 -49.5 54.5 |
| 36hSD<RW | Postcentral Gyrus | L | 3 | 224 | -3.9277 | 53.5 -25.5 49.5 |
| 36hSD<RW | Precuneus | R | 7 | 209 | -4.1685 | 14.5 -64.5 33.5 |
| 36hSD<RW | Precuneus | R | 7 | 128 | -3.8025 | 14.5 -79.5 43.5 |
| 36hSD<RW | Precuneus, Paracentral Lobule | L,R | 5,7 | 230 | -3.8563 | 0.5 -37.5 48.5 |
| 36hSD<RW | Paracentral Lobule | L | N/A | 272 | -5.7939 | -12.5 -18.5 44.5 |
| 36hSD<RW | Paracentral Lobule | L,R | 5 | 162 | -4.0622 | -0.5 -46.5 58.5 |
| 36hSD<RW | Middle Frontal Gyrus | R | 6 | 506 | -4.7206 | 32.5 -4.5 62.5 |
| Recover>RW | Cerebellum Anterior Lobe | R | N/A | 210 | 4.6657 | 11.5 -55.5 -9.5 |
| Recover>RW | Thalamus | L, R | N/A | 1225 | 6.7665 | | -6.5 -33.5 8.5 | | --- | |
| Recover>RW | Caudate Body | R | N/A | 290 | 4.7665 | 13.5 0.5 21.5 |
| Recover>RW | Insula | L | 13 | 306 | 4.9381 | -32.5 2.5 13.5 |
| Recover>RW | Insula | R | 13 | 1547 | 7.4472 | 37.5 -1.5 16.5 |
| Recover>RW | Insula, Inferior Parietal Lobule | L | 13, 40 | 1172 | 6.768 | -41.5 -31.5 21.5 |
| Recover>RW | Superior Parietal Lobule, Inferior Parietal Lobule | L | 7,40 | 1402 | 5.6289 | -32.5 -63.5 47.5 |
| Recover>RW | Inferior Parietal Lobule, Postcentral Gyrus | R | 2,7,40 | 2586 | 5.3461 | 32.5 -50.5 46.5 |
| Recover>RW | Postcentral Gyrus, Inferior Parietal Lobule | L | 2,40 | 434 | 5.2901 | -36.5 -35.5 46.5 |
| Recover>RW | Paracentral Lobule | L,R | 5 | 621 | 4.378 | 10.5 -30.5 49.5 |
| Recover>RW | Precuneus | L | 7 | 115 | 3.5352 | -23.5 -74.5 42.5 |
| Recover>RW | Precuneus | R | 7 | 1006 | 4.5859 | 12.5 -74.5 35.5 |
| Recover>RW | Precuneus | R | 19 | 404 | 3.9733 | 30.5 -77.5 26.5 |
| Recover>RW | Middle Frontal Gyrus | R | 6 | 196 | 4.841 | 24.5 8.5 57.5 |

**Notes:** RW, Rested wakefulness; R, right; L, left; BA, Brodmann’s area; MNI, montreal neurological institute; N/A, Not applicable. The statistical threshold was set at uncorrected voxel threshold of p<0.001 with a minimum cluster threshold of 100 voxels (t=3.1697).
