# Supplementary material for: Plasticity and Susceptibility of Brain Morphometry Alterations to Insufficient Sleep
Source: Front Psychiatry. 2018 Jun 27;9:266. doi: 10.3389/fpsyt.2018.00266 (PMC6030367; doi:10.3389/fpsyt.2018.00266)
Supplement: Supplemental Table 3 — The gray matter volume differences without applying mask method in the 36 h sleep deprivation (SD) study and in the chronic insomnia study. RW, Rested wakefulness; R, right; L, left; BA, Brodmann's area; MNI, montreal neurological institute; N/A, Not applicable; GSs, good sleepers. The statistical threshold was set at family-wise error corrected voxel threshold of p < 0.05 of each time in the 36 h SD study without product with the mask image of main effect, and at uncorrected voxel threshold of p < 0.001 with a minimum cluster threshold of 100 voxels in patients with insomnia. [file Table_3.DOC]

**Plasticity and susceptibility of brain morphometry alterations to insufficient sleep**

**Supplemental Information**

**Supplemental Table 3** The gray matter volume differences without applying mask method in 36h sleep deprivation (SD) study and in chronic insomnia study

| Conditions | Brain regions of peak coordinates | R/L | BA | Voxel size | t-score of peak voxel | MNI coordinates |
| --- | --- | --- | --- | --- | --- | --- |
|  |  |  |  |  |  | X, Y, Z |
| 24h SD>RW | Caudate | R | N/A | 76 | 5.3214 | 10.5 17.5 1.5 |
| 24h SD>RW | Caudate | L | N/A | 68 | 5.4953 | -6.5 18.5 2.5 |
| 24h SD>RW | Superior Frontal Gyrus | R | 10 | 10 | 5.1127 | 26.5 56.5 4.5 |
| 24h SD>RW | Posterior Cingulate, Corpus Callosum | R | 19, 30 | 101 | 5.6732 | 19.5 -50.5 6.5 |
| 24h SD>RW | Cingulate Gyrus, Corpus Callosum | L, R | 23 | 596 | 6.3082 | 2.5 -35.5 22.5 |
| 24h SD>RW | Cingulate Gyrus | R | 23 | 76 | 5.7818 | 12.5 -22.5 36.5 |
| 36h SD>RW | Cerebellum Posterior Lobe | R | N/A | 6 | 5.116 | 47.5 -67.5 -29.5 |
| 36h SD>RW | Caudate | L | N/A | 38 | 5.5553 | -9.5 17.5 -4.5 |
| 36h SD>RW | Postcentral Gyrus | L | 3 | 2 | 5.1249 | -35.5 -28.5 42.5 |
| 36h SD<RW | Inferior Frontal Gyrus, Superior Temporal Gyrus | R | 38 | 69 | -5.4046 | 32.5 9.5 -17.5 |
| 36h SD<RW | Cingulate Gyrus | L | 24 | 68 | -5.7939 | -12.5 -18.5 44.5 |
| Recover>RW | Rectal Gyrus | L | 11 | 47 | 5.5876 | -8.5 33.5 -28.5 |
| Recover>RW | Thalamus | R | N/A | 140 | 5.7342 | 10.5 -31.5 7.5 |
| Recover>RW | Thalamus | L | N/A | 214 | 6.7665 | -6.5 -33.5 8.5 |
| Recover>RW | Cuneus | R | 18 | 274 | 5.5956 | 2.5 -76.5 11.5 |
| Recover>RW | Insula | R | 13 | 655 | 7.4472 | 37.5 -1.5 16.5 |
| Recover>RW | Inferior Parietal Lobule, Insula | L | 13 | 216 | 6.768 | -41.5 -31.5 21.5 |
| Recover>RW | Precentral Gyrus, Postcentral Gyrus | L | 3, 4 | 116 | 5.5874 | -41.5 -20.5 43.5 |
| Recover>RW | Superior Parietal Lobule | L | 7 | 77 | 5.6289 | -32.5 -63.5 47.5 |
| Recover>RW | Inferior Parietal Lobule | R | 7, 40 | 51 | 5.3461 | 32.5 -50.5 46.5 |
| Recover>RW | Parietal Lobe | L | 7 | 60 | 5.3831 | -31.5 -46.5 47.5 |
| Recover>RW | Parietal Lobe | R | 2 | 78 | 5.2702 | 40.5 -33.5 46.5 |
| Recover>RW | Parietal Lobe | L | 40 | 32 | 5.2901 | -36.5 -35.5 46.5 |
| PIs>GSs | Fusiform Gyrus | R | 37 | 227 | 3.6093 | 41.5 -47.5 -21.5 |
| PIs>GSs | Cerebellum Anterior Lobe, Lingual Gyrus | R | N/A | 130 | 3.6429 | 2.5 -60.5 0.5 |
| PIs>GSs | Claustrum, Insula | R | 13 | 185 | 3.5236 | 34.5 -19.5 6.5 |
| PIs>GSs | Superior Temporal Gyrus | R | 22,42 | 1392 | 3.7118 | 70.5 -30.5 17.5 |
| PIs>GSs | Superior Frontal Gyrus | R | 6 | 217 | 3.5821 | 9.5 13.5 66.5 |
| PIs<GSs | Lingual Gyrus | R | 18 | 419 | -3.9209 | 15.5 -92.5 -5.5 |

**Notes:** RW, Rested wakefulness; R, right; L, left; BA, Brodmann’s area; MNI, montreal neurological institute; N/A, Not applicable; PIs, patients with chronic primary insomnia; GSs, good sleepers. The statistical threshold was set at family-wise error corrected voxel threshold of p<0.05 of each time in 36h SD study without product with the mask image of main effect, and at uncorrected voxel threshold of p<0.001 with a minimum cluster threshold of 100 voxels in PIs.
